# Recessive inborn errors of type I IFN immunity in children with COVID-19 pneumonia

**DOI:** 10.1084/jem.20220131

**Published:** 2022-06-16

**Authors:** Qian Zhang, Daniela Matuozzo, Jérémie Le Pen, Danyel Lee, Leen Moens, Takaki Asano, Jonathan Bohlen, Zhiyong Liu, Marcela Moncada-Velez, Yasemin Kendir-Demirkol, Huie Jing, Lucy Bizien, Astrid Marchal, Hassan Abolhassani, Selket Delafontaine, Giorgia Bucciol, COVID Human Genetic Effort, Laurent Abel, Hassan Abolhassani, Alessandro Aiuti, Ozge Metin Akcan, Saleh Al-Muhsen, Fahd Al-Mulla, Gulsum Alkan, Mark S. Anderson, Evangelos Andreakos, Andrés A. Arias, Jalila El Bakkouri, Hagit Baris Feldman, Alexandre Belot, Catherine M. Biggs, Dusan Bogunovic, Alexandre Bolze, Anastasiia Bondarenko, Ahmed A. Bousfiha, Sefika Elmas Bozdemir, Petter Brodin, Yenan Bryceson, Carlos D. Bustamante, Manish J. Butte, Giorgio Casari, John Christodoulou, Roger Colobran, Antonio Condino-Neto, Stefan N. Constantinescu, Megan A. Cooper, Clifton L. Dalgard, Murkesh Desai, Beth A. Drolet, Jamila El Baghdadi, Melike Emiroglu, Emine Hafize Erdeniz, Sara Espinosa-Padilla, Jacques Fellay, Carlos Flores, José Luis Franco, Antoine Froidure, Peter K. Gregersen, Bodo Grimbacher, Belgin Gulhan, Filomeen Haerynck, David Hagin, Rabih Halwani, Lennart Hammarström, James R. Heath, Sarah E. Henrickson, Elena W.Y. Hsieh, Eystein Husebye, Kohsuke Imai, Yuval Itan, Petr Jabandziev, Erich D. Jarvis, Timokratis Karamitros, Adem Karbuz, Kai Kisand, Cheng-Lung Ku, Yu-Lung Lau, Yun Ling, Carrie L. Lucas, Tom Maniatis, Davood Mansouri, László Maródi, Ayse Metin, Isabelle Meyts, Joshua D. Milner, Kristina Mironska, Trine H. Mogensen, Tomohiro Morio, Lisa F.P. Ng, Luigi D. Notarangelo, Antonio Novelli, Giuseppe Novelli, Cliona O'Farrelly, Satoshi Okada, Keisuke Okamoto, Şadiye Kübra Tüter Öz, Tayfun Ozcelik, Qiang Pan-Hammarström, Maria Papadaki, Jean W. Pape, Aslinur Ozkaya Parlakay, Rebeca Perez de Diego, David S. Perlin, Graziano Pesole, Anna M. Planas, Petra Pokorna, Carolina Prando, Aurora Pujol, Lluis Quintana-Murci, Sathishkumar Ramaswamy, Laurent Renia, Igor Resnick, Jacques G. Rivière, Carlos Rodríguez-Gallego, Vanessa Sancho-Shimizu, Anna Sediva, Mikko R.J. Seppänen, Mohammed Shahrooei, Anna Shcherbina, Katerina Slaba, Ondrej Slaby, Andrew L. Snow, Pere Soler-Palacín, Lien De Somer, András N. Spaan, Ivan Tancevski, Stuart G. Tangye, Ahmad Abou Tayoun, Dimitris Thanos, Stuart E. Turvey, K M Furkan Uddin, Mohammed J. Uddin, Diederik van de Beek, François Vermeulen, Donald C. Vinh, Horst von Bernuth, Joost Wauters, Carine Wouters, Aysun Yahsi, Saliha Kanik Yuksek, Mayana Zatz, Pawel Zawadzki, Helen C. Su, Jean-Laurent Casanova, Gulsum Ical Bayhan, Sevgi Keles, Ayca Kiykim, Selda Hancerli, Filomeen Haerynck, Benoit Florkin, Nevin Hatipoglu, Tayfun Ozcelik, Guillaume Morelle, Mayana Zatz, Lisa F.P. Ng, David Chien Lye, Barnaby Edward Young, Yee-Sin Leo, Clifton L. Dalgard, Richard P. Lifton, Laurent Renia, Isabelle Meyts, Emmanuelle Jouanguy, Lennart Hammarström, Qiang Pan-Hammarström, Bertrand Boisson, Paul Bastard, Helen C. Su, Stéphanie Boisson-Dupuis, Laurent Abel, Charles M. Rice, Shen-Ying Zhang, Aurélie Cobat, Jean-Laurent Casanova

**Affiliations:** 1 St. Giles Laboratory of Human Genetics of Infectious Diseases, Rockefeller Branch, The Rockefeller University, New York, NY; 2 Laboratory of Human Genetics of Infectious Diseases, Necker Branch, INSERM U1163, Paris, France; 3 University Paris Cité, Imagine Institute, Paris, France; 4 Laboratory of Virology and Infectious Diseases, The Rockefeller University, New York, NY; 5 Laboratory for Inborn Errors of Immunity, Department of Microbiology, Immunology and Transplantation, KU Leuven, Leuven, Belgium; 6 Laboratory of Clinical Immunology and Microbiology, Intramural Research Program, National Institute of Allergy and Infectious Diseases, National Institutes of Health, Bethesda, MD; 7 Department of Biosciences and Nutrition, Karolinska Institute, Stockholm, Sweden; 8 Research Center for Immunodeficiencies, Pediatrics Center of Excellence, Children’s Medical Center, Tehran University of Medical Sciences, Tehran, Iran; 9 Department of Pediatrics, University Hospitals Leuven, Leuven, Belgium; 10 Yildirim Beyazit University, Ankara City Hospital, Ankara, Turkey; 11 Necmettin Erbakan University, Meram Medical Faculty, Division of Pediatric Allergy and Immunology, Konya, Turkey; 12 Istanbul University-Cerrahpasa, Pediatric Allergy and Immunology, Istanbul, Turkey; 13 Department of Pediatrics (Infectious Diseases), Istanbul Faculty of Medicine, Istanbul University, Istanbul, Turkey; 14 Department of Pediatric Immunology and Pulmonology, Department of Internal Medicine and Pediatrics, Centre for Primary Immunodeficiency Ghent, PID Research Laboratory, Jeffrey Modell Diagnosis and Research Centre, Ghent University Hospital, Ghent, Belgium; 15 Department of Pediatrics, Hôpital de la Citadelle, Liége, Belgium; 16 Pediatric Infectious Diseases Unit, Bakirkoy Dr. Sadi Konuk Training and Research Hospital, University of Health Sciences, Istanbul, Turkey; 17 Department of Molecular Biology and Genetics, Bilkent University, Bilkent-Ankara, Turkey; 18 Department of General Pediatrics, Bicêtre Hospital, Assistance Publique – Hôpitaux de Paris, University of Paris Saclay, Le Kremlin-Bicêtre, France; 19 Biosciences Institute, University of São Paulo, São Paulo, Brazil; 20 A*STAR Infectious Diseases Labs (A*STAR ID Labs), Agency for Science, Technology and Research (A*STAR), Singapore, Singapore; 21 National Centre for Infectious Diseases, Singapore, Singapore; 22 Lee Kong Chian School of Medicine, Nanyang Technological University, Singapore, Singapore; 23 Yong Loo Lin School of Medicine, National University of Singapore, Singapore, Singapore; 24 Tan Tock Seng Hospital, Singapore, Singapore; 25 The American Genome Center, Uniformed Services University of the Health Sciences, Bethesda, MD; 26 Department of Anatomy, Physiology & Genetics, Uniformed Services University of the Health Sciences, Bethesda, MD; 27 Laboratory of Genetics and Genomics, The Rockefeller University, New York, NY; 28 Department of Genetics, Yale University School of Medicine, New Haven, CT; 29 Yale Center for Genome Analysis, Yale School of Medicine, New Haven, CT; 30 School of Biological Sciences, Nanyang Technological University, Singapore, Singapore; 31 Department of Pediatrics, Necker Hospital for Sick Children, Paris, France; 32 Howard Hughes Medical Institute, New York, NY

## Abstract

Recessive or dominant inborn errors of type I interferon (IFN) immunity can underlie critical COVID-19 pneumonia in unvaccinated adults. The risk of COVID-19 pneumonia in unvaccinated children, which is much lower than in unvaccinated adults, remains unexplained. In an international cohort of 112 children (<16 yr old) hospitalized for COVID-19 pneumonia, we report 12 children (10.7%) aged 1.5–13 yr with critical (7 children), severe (3), and moderate (2) pneumonia and 4 of the 15 known clinically recessive and biochemically complete inborn errors of type I IFN immunity: X-linked recessive TLR7 deficiency (7 children) and autosomal recessive IFNAR1 (1), STAT2 (1), or TYK2 (3) deficiencies. Fibroblasts deficient for IFNAR1, STAT2, or TYK2 are highly vulnerable to SARS-CoV-2. These 15 deficiencies were not found in 1,224 children and adults with benign SARS-CoV-2 infection without pneumonia (P = 1.2 × 10^−11^) and with overlapping age, sex, consanguinity, and ethnicity characteristics. Recessive complete deficiencies of type I IFN immunity may underlie ∼10% of hospitalizations for COVID-19 pneumonia in children.

## Teaser

In an international cohort of 112 children hospitalized for moderate to critical COVID-19 pneumonia, we identified 12 children with one of four known recessive inborn errors of type I interferon immunity: X-linked TLR7 and autosomal IFNAR1, STAT2, and TYK2 deficiencies.

## Graphical Abstract

**Figure:**
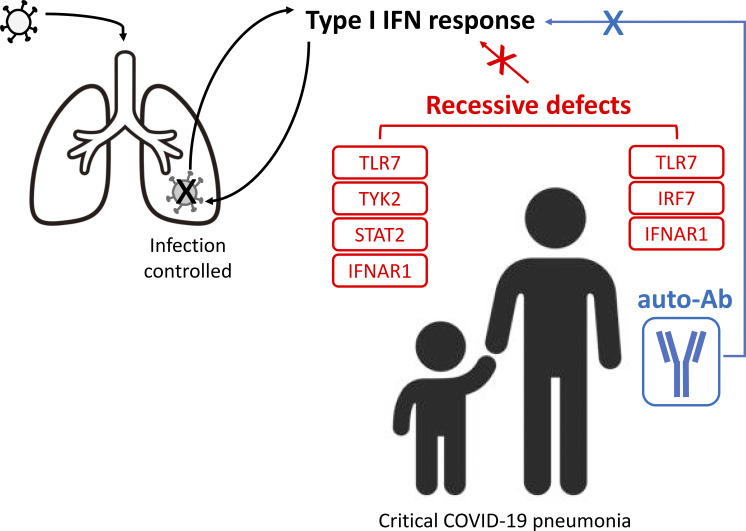

## Introduction

SARS-CoV-2 infection in unvaccinated individuals is silent or mild (i.e., causing a benign upper respiratory tract disease) in ∼80% of cases (Brodin, 2021; Telenti et al., 2021; Zhang et al., 2022). Moderate, nonhypoxemic pneumonia is seen in ∼10% of cases. Hypoxemic pneumonia occurs in ∼10% of cases and can be severe (∼7%, with O_2_ <6 liters/min) or critical (∼3%, with O_2_ >6 liters/min and/or mechanical ventilation). The overall infection fatality rate (IFR) is ∼1%, with significant geographic variations. The risk of death doubles every 5 yr of age, from childhood onward, accounting for >99.9% of patients with critical pneumonia being adults (over 16 yr of age; O’Driscoll et al., 2021). We tested patients for influenza susceptibility genes, and we identified autosomal inborn errors of TLR3-dependent and -independent type I IFN immunity in ∼3% of adults with critical COVID-19 pneumonia, including, surprisingly, autosomal recessive (AR) deficiencies of IFNAR1 or IRF7 in four previously healthy, unrelated adults aged 25–50 yr (Zhang et al., 2020b). AR IRF7 deficiency impairs the production of type I and III IFNs, especially in plasmacytoid dendritic cells (pDCs), which normally constitutively express high levels of IRF7 (Ciancanelli et al., 2015), whereas AR IFNAR1 deficiency impairs cellular responses to type I but not III IFNs, across cell types (Hernandez et al., 2018). Two patients with AR IFNAR1 deficiency, one aged 3 yr and the other aged 13 yr (Abolhassani et al., 2022; Khanmohammadi et al., 2021), and a 3.5-yr-old child with AR TBK1 deficiency (Schmidt et al., 2021) were subsequently reported.

Using an unbiased genetic approach, we also identified X-linked recessive (XR) TLR7 deficiency in 17 male patients aged 7–71 yr with critical COVID-19 pneumonia, accounting for ∼1% of cases in men (Asano et al., 2021). These patients included the only known patient with ataxia-telangiectasia who developed critical disease (Abolhassani et al., 2021). Moreover, 9 (aged 21–57 yr) of the other 19 patients with a proposed diagnosis of TLR7 deficiency (Fallerini et al., 2021; Mantovani et al., 2021; Pessoa et al., 2021; Solanich et al., 2021; van der Made et al., 2020) actually had TLR7 deficiency according to the results of our own biochemical study (Asano et al., 2021). Finally, we found preexisting autoantibodies (auto-Abs) neutralizing type I IFNs in ∼15% of critical cases, with a higher proportion in patients older than 70 yr (Bastard et al., 2021a; Bastard et al., 2020). Human type I IFNs are, therefore, essential for protective immunity to SARS-CoV-2 in the respiratory tract (Casanova and Abel, 2021; Zhang et al., 2022). These findings also incriminated two key cell types governing type I IFN immunity to the virus: respiratory epithelial cells (RECs), which express TLR3 and allow viral replication (Zhang et al., 2022; Zhang et al., 2020b), and pDCs, which express TLR7 and can sense the virus but do not allow viral replication (Asano et al., 2021; Zhang et al., 2022). TLR3 is an endosomal sensor of double-stranded RNA (dsRNA) that governs tonic type I IFN levels in several nonhematopoietic cell types, including RECs (Alexopoulou et al., 2001; Gao et al., 2021), whereas TLR7 is an endosomal sensor of single-stranded RNA (ssRNA; Diebold et al., 2004; Heil et al., 2004; Lund et al., 2004).

The penetrance of AR IFNAR1 and IRF7 deficiencies for critical COVID-19 appears to be complete, whereas that of XR TLR7 deficiency is high, but incomplete, especially in young patients (Asano et al., 2021). Consistently, critical COVID-19 pneumonia is both much less common and much less well understood in children than in adults. There are 15 known inborn errors of type I IFN that are recessively inherited and biochemically complete (Meyts and Casanova, 2021): mutations of *TLR3* (Guo et al., 2011; Zhang et al., 2007), *TICAM1* (Sancho-Shimizu et al., 2011), *UNC93B1* (Casrouge et al., 2006), *TLR7* (Asano et al., 2021), *IRAK4* (Nishimura et al., 2021; Picard et al., 2003), *MYD88* (Giardino et al., 2016; von Bernuth et al., 2008), *IFIH1* (Asgari et al., 2017; Chen et al., 2021; Lamborn et al., 2017), *TBK1* (Schmidt et al., 2021; Taft et al., 2021), *IRF7* (Ciancanelli et al., 2015; Zhang et al., 2020b), *IFNAR1* (Abolhassani et al., 2022; Bastard et al., 2021c; Hernandez et al., 2019), *IFNAR2* (Bastard et al., 2021d; Duncan et al., 2015), *TYK2* (Minegishi et al., 2006; Sarrafzadeh et al., 2020), *STAT1* (Dupuis et al., 2003; Le Voyer et al., 2021), *STAT2* (Freij et al., 2020; Hambleton et al., 2013), and *IRF9* (Bravo Garcia-Morato et al., 2019; Hernandez et al., 2018). Deficiencies of TLR3 and TRIF (encoded by *TICAM1*) disrupt the TLR3 pathway, whereas deficiencies of TLR7, MYD88, and IRAK4 affect the TLR7 pathway. UNC93B deficiency impairs both TLR3 and TLR7 responses. TBK1 deficiency impairs responses to TLR3, TLR7, and IFIH1. Deficiencies of MYD88, IRAK4, UNC93B, and TBK1 also disrupt the TLR8- and TLR9-dependent induction of type I IFNs. Finally, deficiencies of IFNAR1, IFNAR2, TYK2, STAT1, STAT2, and IRF9 impair cellular responses to type I IFN (Casanova and Abel, 2021; Zhang et al., 2022).

These disorders (or, by inference from their milder, dominant form, for autosomal disorders, underlined) are associated with severe viral diseases, including influenza pneumonia (TLR3, IRF7, IRF9, STAT1, STAT2; Alosaimi et al., 2019; Ciancanelli et al., 2015; Hernandez et al., 2018; Le Voyer et al., 2021), COVID-19 pneumonia (TLR3, UNC93B1, TBK1, TRIF, IRF3, IRF7, IFNAR1, IFNAR2, TLR7; Abolhassani et al., 2022; Asano et al., 2021; Schmidt et al., 2021; Solanich et al., 2021; van der Made et al., 2020; Zhang et al., 2020b), rhinovirus or respiratory syncytial virus pneumonia (MDA5, encoded by *IFIH1*; Asgari et al., 2017; Lamborn et al., 2017), herpes simplex virus encephalitis (IFNAR1, STAT1, TLR3, TRIF, TYK2, UNC93B1; Bastard et al., 2021c; Casrouge et al., 2006; Dupuis et al., 2003; Guo et al., 2011; Kreins et al., 2015; Minegishi et al., 2006; Sancho-Shimizu et al., 2011), adverse reactions to measles, mumps, and rubella (MMR) or yellow fever virus (YFV) vaccines (IFNAR1, IFNAR2, STAT1, STAT2; Bastard et al., 2022; Burns et al., 2016; Duncan et al., 2015; Duncan et al., 2022; Hambleton et al., 2013; Hernandez et al., 2019; Moens et al., 2017), enterovirus encephalitis (TLR3, MDA5; Chen et al., 2021), EBV viremia (MYD88; Chiriaco et al., 2019), and human herpesvirus-6 (HHV6) infection (IRAK4; Nishimura et al., 2021). Over the last two decades, 105 children with these disorders have been reported, and many more were probably diagnosed. We tested the hypothesis that these 15 recessive disorders could underlie COVID-19 pneumonia in at least some patients <16 yr of age with no history of inborn errors of immunity (IEIs) enrolled by the COVID Human Genetic Effort (http://www.covidhge.com). We did not enroll patients known to suffer from any of these recessive disorders before having COVID-19 in this study, to prevent bias.

## Results and discussion

### Identification of type I IFN–related candidate genes in children hospitalized for COVID-19 pneumonia

**Figure 1.**
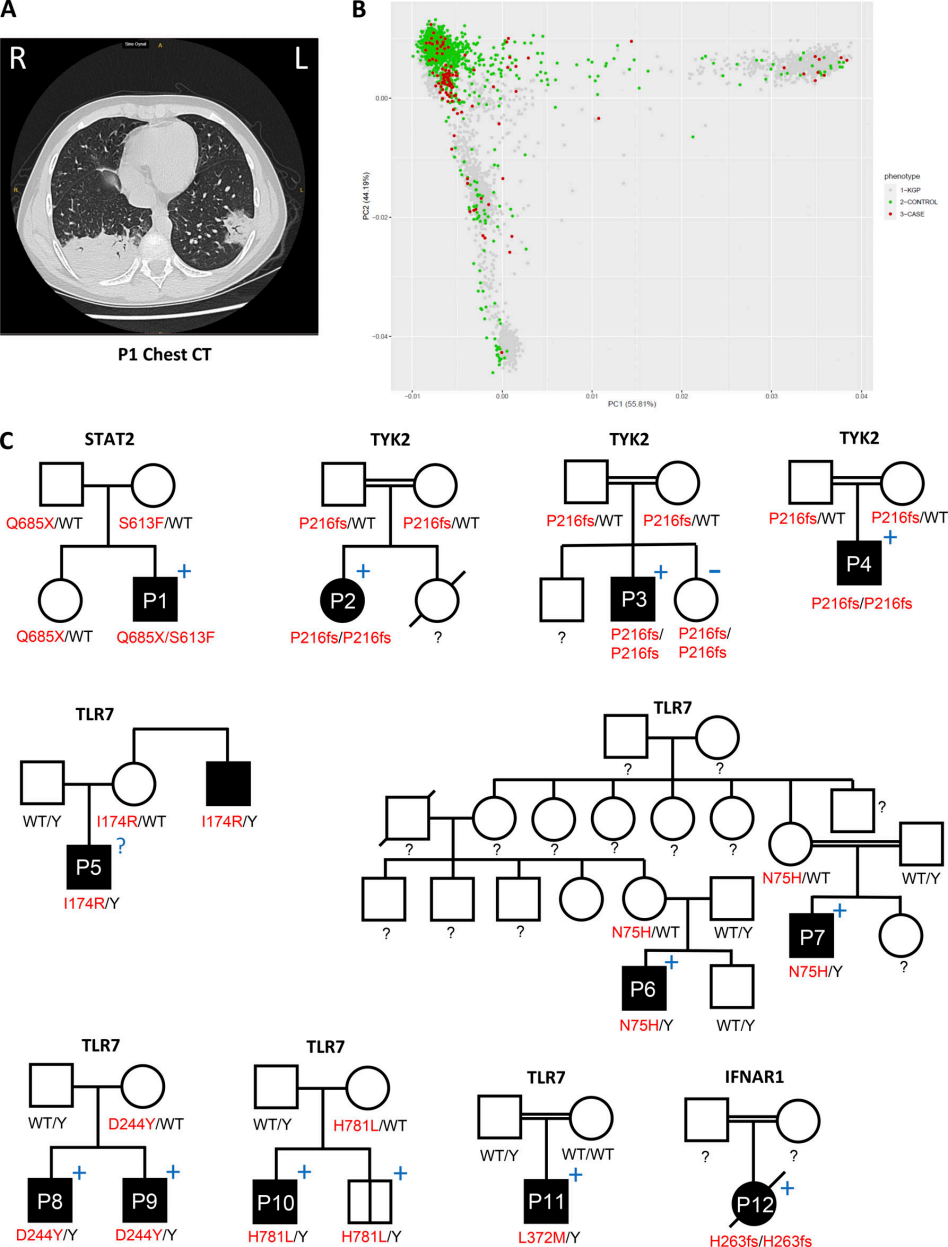
**Recessive inborn errors of the type I IFN pathway underlie life-threatening viral infections. (A)** Chest computed tomography scan on day 6 after disease onset in P1, showing ground-glass opacification and consolidation in both lungs. **(B)** PCA analysis of patients and controls. KGP, 1000 Genomes Project database. **(C)** Pedigrees and familial segregation of the variants identified. Black symbols, patients with moderate to critical COVID-19 pneumonia; symbols with vertical bars, individuals with asymptomatic SARS-CoV-2 infection; blue + and − symbols, seropositive and seronegative for SARS-CoV-2, respectively.

We studied 112 children hospitalized for COVID-19 pneumonia, including 25 with moderate, 15 with severe, and 72 with critical pneumonia. The inclusion criteria were (1) patient under the age of 16 yr; (2) SARS-CoV-2 PCR–positive respiratory tract sample; and (3) radiological proof of COVID-19 pneumonia (Fig. 1 A). Patients already known to have inborn errors of type I IFN immunity were not enrolled in our international cohort. At the time of hospitalization, the patients were living in 14 countries: Belgium (*n* = 2); Brazil (5); Czech Republic (2); Egypt (2); France (10); Hong Kong, China (1); Iran (17); Italy (9); Peru (1); Spain (16); Switzerland (1); Turkey (33); United Arab Emirates (1); and United States (12). Of the 112 patients included in this study, 47 had been reported before (Asano et al., 2021; Bastard et al., 2021a; Bastard et al., 2020; Zhang et al., 2020b): 39 critical cases, 7 severe cases, and 1 moderate case. The candidate genes encoded the type I IFN–inducing dsRNA sensors endosomal TLR3 and cytosolic MDA5 (*IFIH1*), the ssRNA endosomal sensor TLR7; key components of these and other IFN-inducing pathways, including UNC93B1 (for the TLR3 and TLR7 pathways), TRIF (*TICAM1*; for the TLR3 pathway), MYD88 and IRAK4 (for the TLR7 pathway; Onodi et al., 2021); and TBK1 (for TLR3, TLR7, MDA5, and other pathways); the type I IFN receptor chains IFNAR1 and IFNAR2; and key components of their signaling pathway, including TYK2, STAT1, STAT2, IRF7, and IRF9 (Casanova and Abel, 2021; Duncan and Hambleton, 2021; Meyts and Casanova, 2021; Zhang et al., 2020a). *TLR7* is the only X-linked gene, the other 14 genes being autosomal. These are the only known loci in humans for which inborn errors have been reported that (a) are recessive, as opposed to dominant, (b) are biochemically complete (as opposed to partial deficiencies), and (c) impair type I IFN immunity. All reported causal variants at 13 of these 15 loci have a global Genome Aggregation Database (GnomAD, v2.1) minor allele frequency (MAF) <10^−4^ (*IFIH1* and *TLR3* being the exceptions, with causal variants having MAFs of ≤6.7 × 10^−3^ and 1.7 × 10^−3^, respectively). The penetrance of most these genotypes for most associated viral diseases is incomplete. We thus tested the hypothesis that these 15 recessive inborn errors of type I IFN immunity may underlie moderate, severe, or critical COVID-19 in at least some children, who may not necessarily have suffered from other unusually severe viral illnesses before COVID-19.

### Enrichment in rare homozygous or hemizygous variants at the 15 candidate loci

We collected whole-exome sequencing (WES)/whole-genome sequencing (WGS) data for the 112 children hospitalized for COVID-19 pneumonia (hereafter referred to simply as “patients”; 90 WES and 22 WGS) and for 1,224 children and adults with asymptomatic or mild SARS-CoV2 infection (including 82 children aged 6 mo to 16 yr, 90 young adults aged 17–25 yr, and the remaining 1,052 adults >25 yr old, hereafter referred to as “controls”; 714 WES and 510 WGS). The mean age (SD) of the patients was 9.4 (4.6) yr, with a male-to-female ratio of 2.3 (78 boys and 34 girls). By contrast, the controls had a mean age (SD) of 44.8 (19.3) yr (range: 6 mo to 105 yr) with a male-to-female ratio of 0.66 (306 male and 918 female). The patients were of Middle Eastern (*n* = 52), European (*n* = 29), American admixed (*n* = 12), sub-Saharan African (*n* = 10), North African (*n* = 4), South Asian (*n* = 4), and East Asian (*n* = 1) origin, based on principal component analysis (PCA) of their exomes (Fig. 1 B; Belkadi et al., 2016). Among the 112 patients, 26 (23.2%) were considered to have been born to consanguineous parents based on exome homozygosity rates >1% (Belkadi et al., 2016). This rate of consanguinity is higher than the global level of 8.5% (Modell and Darr, 2002). The control individuals were of Middle Eastern (*n* = 153), European (*n* = 895), American admixed (*n* = 77), sub-Saharan African (*n* = 34), North African (*n* = 17), South Asian (*n* = 28), and East Asian (*n* = 20) origin, based on PCA (Fig. 1 B). Among these 1,224 individuals, 75 (6.1%) were considered to have been born to consanguineous parents, based on homozygosity rates >1%. The proportion of patients born to consanguineous parents was significantly higher than that in controls (Fisher’s exact test, P = 3.5 × 10^−8^), but, in terms of absolute numbers, there were more controls (75) than cases (26) born to consanguineous parents. We analyzed the WES or WGS data for these individuals, checking for all 15 recessive inborn errors of type I IFN immunity. We then selected homozygous and potential compound heterozygous nonsynonymous and essential splice site variants at the 14 autosomal loci, and hemizygous variants at the *TLR7* locus. We searched for very rare (MAF <10^−4^) variants among the GnomAD nonsynonymous or splice-site variants with a combined annotation-dependent depletion score greater than the gene-specific mutation significance cutoff, for the 15 genes (Itan et al., 2016). We also searched for rare homozygous or hemizygous copy number variants for the same genes (Bigio et al., 2021; Chen et al., 2016). A burden test adjusted for sex and the first five principal components for the 15 loci revealed highly significant enrichment in rare homozygous, potential compound heterozygous, or hemizygous variants in patients relative to controls (P = 1 × 10^−8^; odds ratio = 26.6; 95% confidence interval = 7.3–96.6), with 12 carriers among the patients (10.7%, including four homozygotes, one potential compound heterozygote, and seven hemizygotes) vs. three (0.25%, two homozygotes, one hemizygote) among the controls. The three carriers among the controls were 44, 50, and 53 yr old. Restricting the analysis to predicted loss-of-function variants (pLOF) also revealed significant enrichment in such variants, with four carriers among the patients (3.5%) and none among the controls (P = 1.4 × 10^−4^). A similar result was obtained when the analysis was performed on pLOF variants without restriction on the basis of MAF. TLR7 deficiency has already been reported to underlie critical COVID-19 in adults (Asano et al., 2021). We therefore also analyzed enrichment, focusing on the 14 autosomal genes, and found a significant enrichment in rare pLOF (1.4 × 10^−4^) or rare pLOF and missense variants (P = 9.6 × 10^−4^). Finally, the enrichment analyses performed on homozygous synonymous variants or on heterozygous nonsynonymous and essential splice variants with MAF <10^−4^ for the 15 loci were not significant (P = 0.05 and 0.79, respectively), indicating that our ethnicity-adjusted burden test was well calibrated.

### Candidate genotypes detected by exome and genome sequence analyses

Our analysis led to the identification of 12 unrelated children with COVID-19 pneumonia, each of whom carries a biallelic or hemizygous variant of one of the 15 candidate genes (Table 1 and Fig. 1 C), as confirmed by Sanger sequencing. One of the patients had two compound heterozygous variants of *STAT2* (S613F and Q685X; the compound heterozygosity of the two mutations was confirmed by family segregation analysis), and three unrelated patients had the same homozygous missense variant of *TYK2* (P216fs*14; AR inheritance was confirmed for all three patients by family segregation analysis). Seven patients had hemizygous missense variants of *TLR7* (one newly identified patient with I174R and six previously described patients with N75H, D244Y, L372M, and H781L; Abolhassani et al., 2021; Asano et al., 2021), and one patient had a large homozygous genomic deletion in *IFNAR1* (H263fs*14, 4,394-bp deletion encompassing exons 7–8 of the *IFNAR1* gene; chr21: 34,719,302–34,723,696, GRCh37–hg19; Abolhassani et al., 2022). The 12 patients originated from 10 kindreds and four countries (Belgium, Russia, Iran, and Turkey; Table 1). Eight of the 12 patients were tested for auto-Abs against type I IFNs, and all were negative (Bastard et al., 2021a; Bastard et al., 2020). We also identified one control with a hemizygous missense variant of *TLR7* (H782D), another with a homozygous missense variant of *IFIH1* (Q415K), and a third with a homozygous missense variant of *IRF7* (L128M). These three controls originated from three different countries: Brazil, France, and Singapore (Table 1). We also screened our sick children for very rare (MAF <10^−4^ according to gnomAD database) homozygous pLOF variants of the 337 genes known to underlie AR or XR IEIs (Tangye et al., 2020; Tangye et al., 2021). We did not identify additional candidate recessive defects. We further screened the full list of 452 IEI genes (including those underlying only dominant disorders) for very rare (MAF <10^−4^) pLOF variants. There was no significant global or gene-specific enrichment in cases vs. controls in tests of a dominant model.

**Table 1.** Genetic, immunological, and clinical description of pediatric patients with recessive inborn errors of type I IFN immunity and COVID-19 pneumonia

Patient	Gene			Gender	Age (yr)	Ethnicity/residence	COVID-19 pneumonia severity	Systemic inflammation	Other viral infections	Other infections and clinical history	Outcome	Publication
P1	*STAT2*	AR	S613F/Q685X (LOF/LOF)	M	12	Middle East/Turkey	Critical	Yes	Aseptic meningitis and Kawasaki disease after MMR vaccination at the age of 1 yr; recurrent severe influenza pneumonia requiring hospitalization since the age of 2 yr		Survived	This report
P2	*TYK2*	AR	P216fs/P216fs (LOF)	F	2	Middle East/Turkey	Moderate		Hospitalized for infection of VZV vaccine at the age of 2 yr	Hospitalized for sepsis during the neonatal period; Admitted to ICU twice for fever and dyspnea before 5 mo old, diagnosed with Kawasaki disease at the age of 11 mo	Survived	This report
P3	*TYK2*	AR	P216fs/P216fs (LOF)	M	4	Middle East/Turkey	Critical		Admitted to ICU for influenza pneumonia at 3 yr; no adverse reaction to MMR vaccination	Hospitalized for sepsis during the neonatal period	Survived	This report
P4	*TYK2*	AR	P216fs/P216fs (LOF)	M	9	Middle East/Turkey	Critical		No adverse reaction to MMR vaccination	Recurrent bronchitis requiring hospital admission and inhaler therapy	Survived	This report
P5	*TLR7*	XR	I174R/Y (LOF)	M	12	Europe/Belgium	Severe		No adverse reaction to MMR vaccination		Survived	This report
P6	*TLR7*	XR	N75H/Y (LOF)	M	7	Middle East/Turkey	Severe		No adverse reaction to MMR vaccination		Survived	Asano et al. (2021)
P7	*TLR7*	XR	N75H/Y (LOF)	M	12	Middle East/Turkey	Severe		No adverse reaction to MMR vaccination		Survived	Asano et al. (2021)
P8	*TLR7*	XR	D244Y/Y (LOF)	M	13	Middle East/Turkey	Critical		No adverse reaction to MMR vaccination		Survived	Asano, et al. (2021)
P9	*TLR7*	XR	D244Y/Y (LOF)	M	5	Middle East/Turkey	Moderate		No adverse reaction to MMR vaccination		Survived	Asano et al. (2021)
P10	*TLR7*	XR	H781L/Y (LOF)	M	13	Middle East/France	Critical		No adverse reaction to MMR vaccination		Survived	Asano et al. (2021)
P11^a^	*TLR7*	XR	L372M/Y (LOF)	M	7	Middle East/Iran	Critical		No adverse reaction to MMR vaccination	Recurrent fever, upper respiratory tract infections and otitis media, pneumonia, since the age of 1 yr; diagnosed with failure to thrive, splenomegaly, anemia, and thrombocytopenia, osteomyelitis of the hip, since the age of 4 yr; diagnosed with hyper IgM syndrome since the age of 5 yr, and received IVIG since then	Survived	Asano et al. (2021); Abolhassani et al. (2021)
P12	*IFNAR1*	AR	H263fs/H263fs (LOF)	F	3	Middle East/Iran	Critical	Yes	No adverse reaction to MMR vaccination	Chronic severe chronic sinusitis and oral thrush since the age of 8 mo; severe mucormycosis of the nose and paranasal sinuses since the age of 2 yr	Deceased	Abolhassani et al. (2022)
C1	*TLR7*	XR	H782D/Y (Neutral)	M	53	Europe/Brazil	Asymptomatic					
C2	*IFIH1*	AR	Q415K/Q415K (Neutral)	F	50	North Africa/France	Asymptomatic					
C3	*IRF7*	AR	L128M/L128M (Neutral)	M	44	Southeast Asia/Singapore	Asymptomatic					

M, male; F, female.

a Patient also carries a homozygous deleterious *ATM* mutation (Y2371X).

### Autosomal and XR type I IFN deficiencies in 12 children

**Figure 2.**
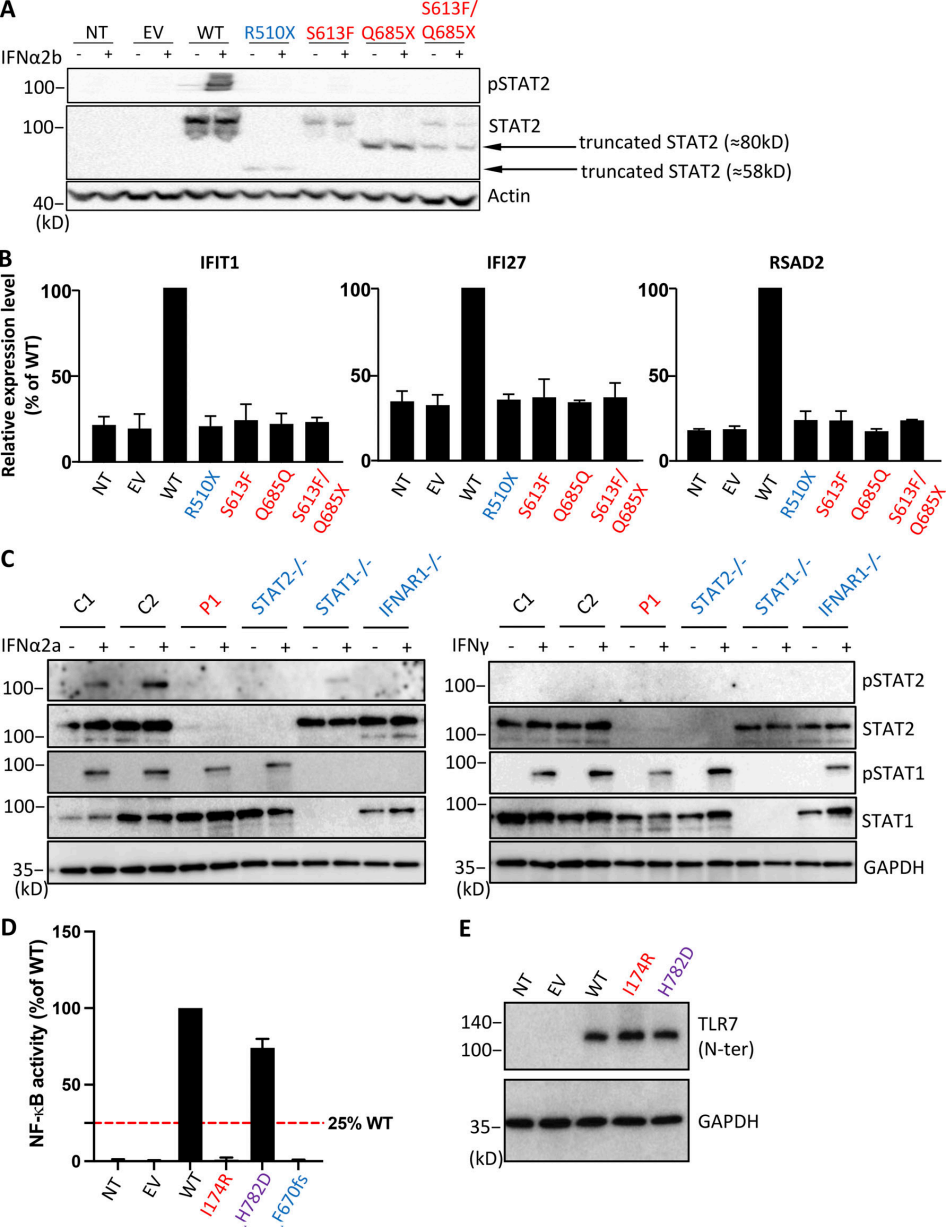
**Novel deleterious variants of *STAT2* and *TLR7* underlie life-threatening COVID-19 in children. (A)** STAT2 protein levels in HEK293T cells, with and without transfection with WT or mutant *STAT2* plasmids, as assessed by Western blotting. The known LOF variant R510X served as an LOF control. **(B)** ISG induction upon stimulation with IFN-α2b, in STAT2-deficient U2A fibrosarcoma cells with and without transfection with the WT or *STAT2* variants. qPCR results were normalized against WT. The known LOF variant R510X served as an LOF control. Experiments were repeated twice. **(C)** Phosphorylated STAT1 (p-STAT1) and p-STAT2 levels following stimulation with IFN-α2a or IFN-γ, as assessed by Western blotting, in SV40 fibroblasts from P1 and previously published patients with AR complete IFNAR1, STAT1, or STAT2 deficiencies and cells from two healthy controls (C1 and C2). **(D)** HEK293T cells were transfected with TLR7 variants, the firefly luciferase gene regulated by NF-κB, and the constitutively expressed *Renilla* luciferase gene, and were then stimulated with the TLR7 agonist R848. Firefly luciferase activity levels were first normalized against *Renilla* luciferase activity, and then against WT activity. The known LOF variant F670fs served as an LOF control. Experiments were repeated four times. Error bars indicate the SD of repeats. **(E)** HEK293T cells were transfected with *TLR7* variants, and protein levels were assessed by Western blotting.

Three of the five patients not previously reported, P2, P3, and P4, carried the same *TYK2* variant (P216Rfs*14), which had already been shown experimentally to be loss-of-expression in cells from patients (Fuchs et al., 2016). We confirmed that this variant was LOF (not depicted). By contrast, the two *STAT2* variants carried by P1 and the TLR7 variant carried by P5 have yet to be studied. We first tested the *STAT2* variants. Following transient overexpression in HEK293T cells, S613F was detected at a molecular weight (MW) similar to that of WT STAT2 (113 kD), whereas Q685X had a lower MW of ∼80 kD; both these variants resulted in the production of only small amounts of protein (Fig. 2 A). Moreover, in HEK293T cells expressing the S613F or Q685X variant, IFN-α2a stimulation did not induce the phosphorylation of STAT2 (pSTAT2), as in cells expressing the known LOF STAT2 variant R510X, but not those expressing WT STAT2 (Fig. 2 A). We then transiently transfected the STAT2-deficient fibrosarcoma cell line U6A with *STAT2* variants to investigate their transcriptional activity. Upon IFN-α2a stimulation, cells transfected with WT STAT2 displayed an induction of transcription for three classical IFN-stimulated genes (ISGs; *IFIT1*, *IFI27*, and *RSAD2*), whereas cells transfected with the patient’s variants and the LOF variant R510X did not (Fig. 2 B). Thus, biochemical tests of the two *STAT2* variants from P1 indicated that both were LOF. We tested simian virus 40–transformed fibroblasts (SV40 fibroblasts) from P1. The phosphorylation of STAT2 (pSTAT2), but not that of STAT1, in response to stimulation with IFN-α2b was abolished, whereas the response to IFN-γ remained intact (Fig. 2 C). Finally, we assessed the expression and function of P5’s *TLR7* variant following transient overexpression in HEK293T cells. The I174R variant of TLR7 failed to activate the NF-κB luciferase reporter when cells were stimulated with the TLR7 agonist R848, as observed for the known LOF TLR7 variant F670fs (Fig. 2 D), although the mutant protein was produced in normal amounts at the expected MW (Fig. 2 E). We can, therefore, conclude that P5’s *TLR7* variant is LOF. These data suggest that P1 has AR STAT2 deficiency and that P5 has XR TLR7 deficiency. The first five patients (P1–P5) reported here, thus, had recessive complete inborn errors of type I IFN immunity. Our findings implicate AR STAT2 and TYK2 deficiencies as new genetic etiologies of COVID-19 pneumonia. Together with the seven previously reported TLR7-deficient (P6–P11) and IFNAR1-deficient (P12) children with COVID-19 pneumonia (Abolhassani et al., 2021; Asano et al., 2021), these five patients bring us to a total of 12 patients (10.7%) in our pediatric COVID-19 cohort with recessive complete inborn errors of type I IFN immunity, including 9.0% of boys for XR traits and 4.5% of children with AR traits. As a means of obtaining a better estimate of this latter proportion, we screened for pairs of individuals with first- to third-degree relationships (kinship ≥0.0442), using the appropriate option of King software (Manichaikul et al., 2010) to estimate the pairwise relatedness between patients. We found two additional pairs of distant relatives in the patient cohort (besides P6/7 and P8/9), and 33 in the control cohort. after the exclusion of one sample per pair, we estimated the frequency of AR type I IFN IEI in our pediatric cohort at 9.3% (10 of 108 unrelated patients).

### Demographic and clinical features of the 12 patients

Five of the 12 patients (P1–P5) are newly described children aged 1.5–13 yr, living in Turkey (P1–P4) and Belgium (P5). All five developed COVID-19 pneumonia of various degrees of severity, from moderate to critical (Table 1), and three had histories of other severe viral diseases before COVID-19 pneumonia, which had not been explored genetically and immunologically. The other seven patients (P6–P12) are previously described children aged 3–13 yr, living in Turkey (P6–P9), France (P10), and Iran (P11 and P12; Abolhassani et al., 2022; Abolhassani et al., 2021; Asano et al., 2021). All seven developed COVID-19 pneumonia of various degrees of severity, from moderate to critical. P11 and P12 had histories of bacterial and fungal infections (Table 1).

P1 was a 12-yr-old Turkish boy, born to healthy non-consanguineous parents. He is compound heterozygous for *STAT2* LOF mutations. He was admitted to the hospital for severe COVID-19 pneumonia (Fig. 1 A) followed by cold agglutinin-mediated autoimmune hemolytic anemia and other mucocutaneus presentations, leading to an initial diagnosis of multisystem inflammatory syndrome in children. P2 (female), P3 (male), and P4 (male) were unrelated 2-, 4-, and 9-yr-old children, each born to a different set of consanguineous Turkish parents. All three are homozygous for the same *TYK2* LOF mutation. P2 was hospitalized for moderate COVID-19 pneumonia but did not require oxygen therapy. P3 and P4 were both admitted to the intensive care unit (ICU) for critical COVID-19 pneumonia requiring high-flow oxygen therapy. P5 is a 12-yr-old European boy living in Belgium who presented with severe pneumonia. He is hemizygous for a *TLR7* LOF mutation (Table 1). The six previously reported TLR7-deficient children (P6–P11) were aged 5–13 yr and were living in Turkey, France, and Iran. They had moderate (one child), severe (two children), or critical (three children) COVID-19 pneumonia, and all survived (Table 1 and Fig. 1 B; Abolhassani et al., 2021; Asano et al., 2021). Finally, the previously reported IFNAR1-deficient child (P12) was a 3-yr-old girl who died from critical COVID-19 (Abolhassani et al., 2022). Moderate to critical COVID-19 pneumonia was a clinical presentation common to the 12 patients. All 11 patients tested mounted normal anti-S antibody responses after infection (Fig. 1 C). These findings suggest that biallelic or hemizygous LOF variants of *IFNAR1*, *STAT2*, *TLR7*, or *TYK2* underlay the COVID-19 pneumonia in these children.

### Penetrance of these recessive defects

We performed Sanger sequencing on family members for whom samples were available. We identified three individuals carrying the same genotype as the index patients (Fig. 1 B). The first, P5’s 52-yr-old maternal uncle, is hemizygous for the same *TLR7* mutation and suffered from critical COVID-19 pneumonia requiring ICU admission and intubation. By contrast, both of P5’s parents had mild COVID-19 that did not require hospitalization (Fig. 1 B). The second was the 8-yr-old younger sibling of P10, who carried the same *TLR7* genotype and had asymptomatic SARS-CoV-2 infection. The third was the 3-yr-old younger sister of P3, who carries the same genotype. She had a history of critical influenza pneumonia that required intubation and ventilation, as well as disseminated varicella. However, she was not infected with SARS-CoV-2 and she has remained seronegative to date (Fig. 1 C). The other sequences were consistent with a recessive trait, with relatives of index cases not hemizygous or homozygous for the pathogenic variant at any of the other three loci. Penetrance for COVID-19 pneumonia was, therefore, complete in the families with AR STAT2, TYK2, and IFNAR1 deficiencies reported here, but incomplete in at least one family with XR TLR7 deficiency, consistent with a previous report (Asano et al., 2021). The benign infection observed in the young TLR7-deficient relative of P10, and the critical diseases observed in P5’s uncle are consistent with our previous findings and suggest that tonic levels of type I IFN in the blood and tissues, which decrease with age (Bartleson et al., 2021; Loske et al., 2021; Pierce et al., 2020; Pierce et al., 2021; Schultze and Aschenbrenner, 2021; Shaw et al., 2013; Splunter et al., 2019; Stark and Darnell, 2012; Zhang et al., 2022), can modify the clinical impact of TLR7 deficiency. The development of COVID-19 pneumonia in a child with STAT2 deficiency is not surprising, given previous reports of critical pneumonia in patients with IFNAR1 or IRF7 deficiency (Zhang et al., 2020b). These defects probably display high, if not complete, penetrance for critical COVID-19. The occurrence of critical pneumonia in three unrelated patients with TYK2 deficiency is more surprising. Indeed, TYK2 deficiency impairs, but does not abolish, cellular responses to type I IFNs (Boisson-Dupuis et al., 2018). More TYK2-deficient patients infected with SARS-CoV-2 need to be diagnosed to estimate the corresponding penetrance.

### Biallelic variants at the same loci in subjects with asymptomatic or mild infection

**Figure 3.**
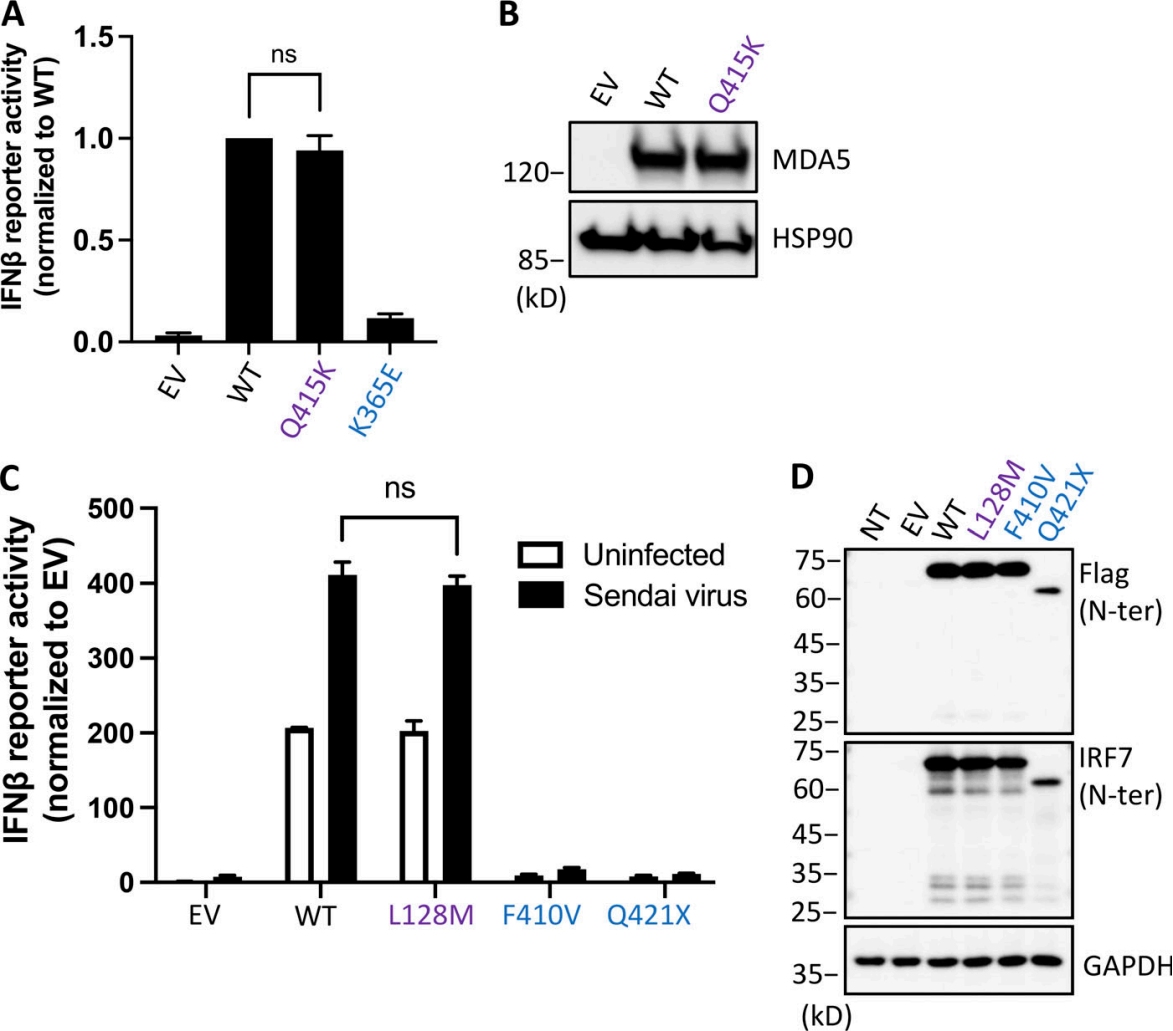
**Functional tests of biallelic variants identified in the control cohort. (A)** HEK293T cells were transfected with MDA5 variants, the firefly luciferase gene regulated by IFN-β, and the constitutively expressed *Renilla* luciferase gene for 24 h and were then stimulated with 2 μg/ml poly(I:C) with Lipofectamine. Firefly luciferase activities were first normalized against *Renilla* luciferase activity and then against WT values. The known LOF variant K365E served as an LOF control. EV, empty vector. Experiments were repeated three times. Error bars indicate the SD of repeats. **(B)** HEK293T cells were transfected with MDA5 variants, and protein levels were assessed by Western blotting. **(C)** HEK293T cells were transfected with IRF7 variants, the firefly luciferase gene regulated by IFN-β, and the constitutively expressed *Renilla* luciferase gene for 24 h and were then stimulated with Sendai viruses or left unstimulated. Firefly luciferase activity was first normalized against *Renilla* luciferase activity and then against unstimulated EV. Two known LOF variants, F410V and Q421X, served as LOF controls. Experiments were repeated three times. Error bars indicate the SD of repeats. **(D)** HEK293T cells were transfected with IRF7 variants, and protein levels were assessed by Western blotting.

We investigated the hemizygous *TLR7* variant (H782D), and the homozygous *IFIH1* (Q415K) and *IRF7* (L128M) variants, which were found in three infected adult controls. We assessed the production and activity of the proteins encoded by the three gene variants following transient overexpression in HEK293T cells. The H782D TLR7 protein had the expected MW and was produced in normal amounts. It also activated the NF-κB luciferase reporter, like WT TLR7, following stimulation with the TLR7 agonist R848 (Fig. 2, D and E). The Q415K MDA5 protein had the expected MW and was produced in normal amounts. It also activated the IFN-β luciferase reporter, like WT MDA5, following intracellular poly(I:C) stimulation (Fig. 3, A and B). Finally, the L128M IRF7 protein was produced at the expected MW and in normal amounts. It activated the IFN-β luciferase reporter normally, with or without Sendai virus stimulation (Fig. 3, C and D). Consequently, the enrichment analysis restricted to biochemically deleterious genotypes was even more significant for the 15 loci (P = 1.9 × 10^−11^), for the 14 autosomal loci without *TLR7* (P = 2.1 × 10^−5^), and remained significant after exclusion of the four pairs of related patients (P = 5.8 × 10^−7^). We found that 12 of 112 children with COVID-19 pneumonia had an AR or XR complete deficiency due to one of four of the 15 known recessive inborn errors of type I IFN immunity. None of the 15 recessive defects were found in 1,224 patients infected with SARS-CoV-2 who did not develop pneumonia. This significant enrichment, together with the known viral infection phenotypes of these four inborn errors (Abolhassani et al., 2022; Abolhassani et al., 2021; Bastard et al., 2021c; Duncan et al., 2015; Freij et al., 2020; Gothe et al., 2020; Hambleton et al., 2013; Hernandez et al., 2019; Kilic et al., 2012; Kreins et al., 2015; Minegishi et al., 2006; Moens et al., 2017; Nemoto et al., 2018; Sarrafzadeh et al., 2020) and the essential role of type I IFNs in protective immunity to SARS-CoV-2 (Asano et al., 2021; Bastard et al., 2021a; Bastard et al., 2020; Zhang et al., 2020b) suggest that these 12 children had COVID-19 pneumonia because of these recessive deficiencies of type I IFN immunity.

### Estimated cumulative frequency of recessive deficiencies in the general population

We finally estimated the cumulative frequency of pLOF variants or pLOF homozygosity at the 15 loci using GnomAD (v2.1). We found a cumulative frequency of pLOF variants of 0.031 and a cumulative frequency of pLOF homozygosity (including hemizygosity for *TLR7*) of 2 × 10^−4^. When the analysis was restricted to the four genes with identified patients (*IFNAR1*, *STAT2*, *TLR7*, and *TYK2*), the cumulative frequency of recessive deficiency, based on the conservative estimate of pLOF variants, was 4 × 10^−5^ for a cumulative MAF of 0.002. Patients with these defects are more likely to be challenged with live attenuated viral vaccines (e.g., first dose of MMR at 12 mo) and common viral infections (e.g., HSV-1 and influenza virus) before exposure to SARS-CoV-2. Only TLR7 deficiency has not been associated with other common viral infections, potentially accounting for the higher proportion of TLR7-deficient patients in our cohort than of patients with other recessive defects. Indeed, three of the five patients without TLR7 deficiency in our cohort had survived severe viral infections requiring hospitalization or intensive care before COVID-19 (P1, infection with MMR vaccine and influenza; P2, infection with MMR vaccine; and P3, influenza), whereas none of the TLR7-deficient patients had been hospitalized for viral infections before COVID-19 infection (Table 1). The viral illnesses in P1, P2, and P3 had not led to genetic and immunological studies. There is currently no accurate estimate of the incidence of critical pneumonia in SARS-CoV-2–infected children, but it is likely to be ∼0.01%, based on an IFR in children of ∼0.001%. The estimated cumulative frequency of recessive deficiency due to pLOF variants of the four genes is consistent with AR or XR inborn errors of type I IFN immunity being causal for COVID-19 pneumonia, with complete (e.g., probably IFNAR1 and STAT2 deficiencies) or incomplete (e.g., TLR7 and perhaps TYK2 deficiencies) penetrance, depending on the locus. Recessive defects of other loci governing type I IFN immunity may also be found in other children.

### Enhanced SARS-CoV-2 replication in STAT2- and TYK2-deficient patients’ cells

**Figure 4.**
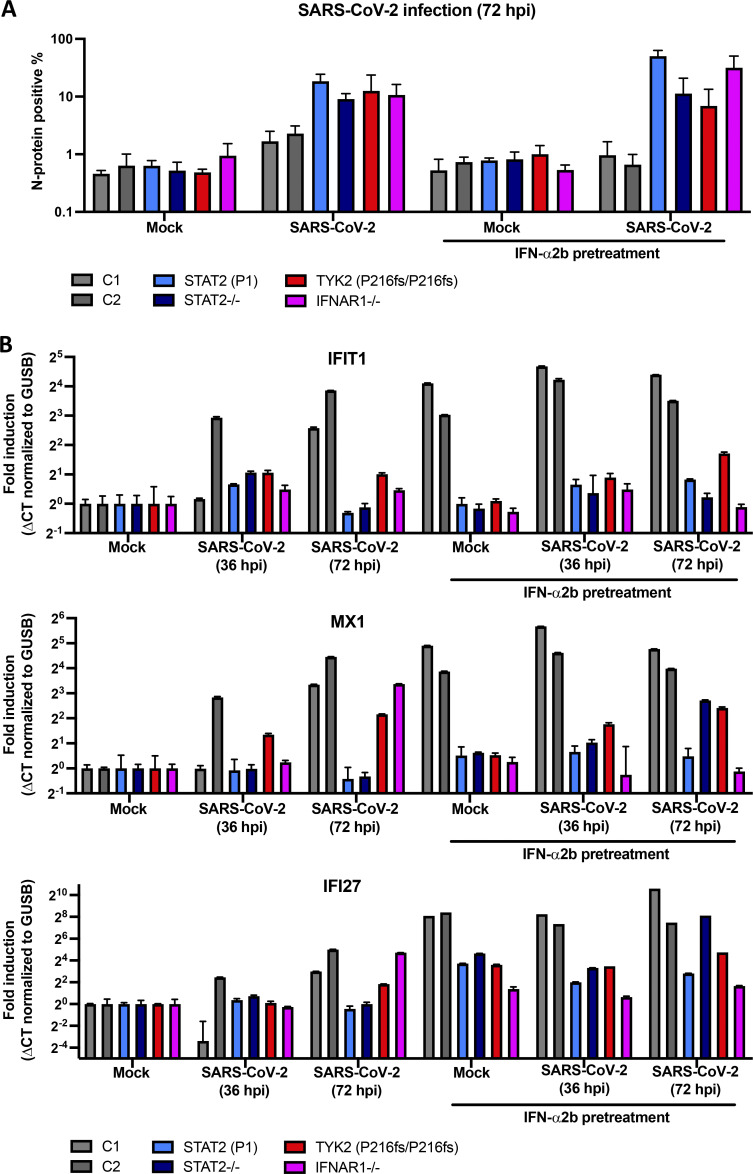
**SARS-CoV-2 infection in patient cells. (A)** Patient SV40 fibroblasts expressing ACE2 were pretreated with 1,000 IU/ml IFN-α2b or left untreated for 16 h and were then infected with SARS-CoV-2 for 72 h. N-protein and cell nuclei were stained with specific anti-N-protein antibody and Hoechst 33342, respectively. The percentage of cells positive for N-protein was determined automatically. We tested SV40 fibroblasts from P1 and a patient with the same genotype as P2–P4 (homozygous for P216fs*14). SV40 fibroblasts from a patient with complete STAT2 deficiency (G522R/R506*) and another patient with complete IFNAR1 deficiency (homozygous V225fs) served as positive controls, whereas SV40 fibroblasts from two healthy donors (C1 and C2) served as negative controls. Experiment was done once with four technical replicates. Error bars indicate the SD of four technical repeats. **(B)** The fold-induction of ISGs (IFIT1, MX1, and IFI27) was determined by qPCR, with normalization against the housekeeping gene GUSB, and then untreated mock infection, with experiments performed in parallel with A. Experiments were repeated twice. Error bars indicate the SD of the repeats.

We previously showed that the SV40-transformed fibroblasts (SV40 fibroblasts) of TLR3-, IRF7-, and IFNAR1-deficient patients cannot control SARS-CoV-2 infection normally in vitro (Zhang et al., 2020b), and that the pDCs of IRAK4-, UNC93B-, and TLR7-deficient patients cannot induce type I IFNs normally when challenged with SARS-CoV-2 in vitro (Asano et al., 2021; Onodi et al., 2021). We hypothesized that cells from STAT2- and TYK2-deficient patients might also be unable to restrict the replication of SARS-CoV-2 normally. We transduced SV40 fibroblasts with ACE2, which rendered them permissive to SARS-CoV-2 infection. We infected SV40 fibroblasts from a TYK2-deficient patient with the same genotype as P2–P4 (homozygous for P216fs*14) with SARS-CoV-2. We also infected SV40 fibroblasts from P1, a STAT2-deficient patient. We measured the intracellular expression of the viral nucleocapsid protein (N-protein) as an indicator of viral replication. Using healthy donor cells as positive controls, and cells from previously diagnosed patients with complete IFNAR1 and STAT2 deficiencies as negative controls, we found that cells from STAT2- and TYK2-deficient patients did not control viral replication normally (Fig. 4 A). Moreover, pretreatment of the cells with IFN-α2b blocked viral infection in healthy donor cells but not in patient cells (Fig. 4 A). We then measured ISGs (IFIT1, MX1, and IFI27) induction in the SARS-CoV-2–infected cells by quantitative RT-PCR (qPCR). We found that cells from STAT2- and TYK2-deficient patients failed to induce ISG production in response to SARS-CoV-2 infection or IFN-α2b pretreatment (Fig. 4 B). Thus, cells from STAT2- and TYK2-deficient patients were unable to control SARS-CoV-2 infection in a type I IFN-dependent manner in vitro. These findings further suggest that these deficiencies were causal for COVID-19 pneumonia in patients with either disorder.

### Concluding remarks

Our findings suggest that three of the 14 known AR inborn errors of type I IFN immunity underlie COVID-19 pneumonia in ∼4% of children. A strong enrichment was observed for very rare (MAF <10^−4^) pLOF and missense variants, and for pLOF variants regardless of their MAF. A study of missense variants with higher frequencies might increase this proportion, but this would require a biochemical characterization of all variants in the general population, as previously performed for *TLR7* (Asano et al., 2021). We also found XR TLR7 deficiency in ∼6% of children and 8.9% of boys with pneumonia. We provide evidence that recessive and complete defects at these four loci can underlie ∼10% of cases of COVID-19 pneumonia in hospitalized children, but the possible role of the other 11 loci in COVID-19 pneumonia remains unclear. Recessive complete defects at the *IFNAR2*, *STAT1*, and *IRF9* (Levy et al., 2021) loci would also probably be causal, given the identification of patients with IFNAR1, TYK2, STAT2, and IRF7 deficiencies (Abolhassani et al., 2022; Khanmohammadi et al., 2021; Zhang et al., 2020b; this report), as would defects at the *TBK1* locus, given the previous report (Schmidt et al., 2021), and defects at the *UNC93B1*, *MYD88*, and *IRAK4* loci, given the identification of patients with TLR7 deficiency (Asano et al., 2021). It is also probable that recessive defects of MDA5, TLR3, and TRIF may underlie COVID pneumonia in children. The high proportion of TLR7-deficient patients in the pediatric COVID-19 pneumonia cohort may reflect the narrow spectrum of viral infections in these patients and the XR inheritance of the disease. Accordingly, the apparent lack of IFNAR2, IRF9, and MDA5 defects may result from other life-threatening infections at an earlier stage of the lives of affected patients and their AR inheritance, whereas the apparent lack of AR STAT1, TLR3, and TRIF defects may also be due to the greater rarity of these defects, due in part to the occurrence of an AD form of these genetic defects.

The prevalence of AR disorders was 4.6% in the 108 unrelated children with COVID-19 pneumonia, with a significantly higher prevalence in patients born to consanguineous parents (with homozygosity rates >1%) than in patients not born to consanguineous parents (16 vs. 1.2%, Fisher’s exact test, P = 0.01), and 5.7% in the 70 unrelated children with critical COVID-19 pneumonia, much higher than the 0.6% of critical COVID-19 pneumonia cases in adults over the age of 16 yr or the 0.8% of patients with critical COVID-19 pneumonia ages 16–60 yr from the same international cohort (Zhang et al., 2020b). In addition, XR TLR7 deficiency was identified in 7% of the 71 unrelated male children with COVID-19 pneumonia, with no significant difference between male patients born to consanguineous parents and male patients born to non-consanguineous parents (20 vs. 7.7%, Fisher’s exact test, P = 0.27), and 6.1% of 49 unrelated male children with critical COVID-19 pneumonia, vs. 1.3% of adult males with critical pneumonia ages 16–60 yr (Asano et al., 2021). This higher proportion probably results from (a) the small number of patients with these inborn errors reaching adulthood undiagnosed, and (b) other risk factors, such as auto-Abs against type I IFN, which increase with age (Manry et al., 2022). However, the clinical penetrance of these four recessive type I IFN deficiencies for COVID-19 pneumonia probably increases with age and probably depends on the deficiency. It is predicted to be higher for STAT2 deficiency (unresponsive to both type I and III IFNs) than for IRF7 (inability to produce type I and III IFNs other than IFN-β) and IFNAR1 deficiencies (unresponsive to type I IFNs), and TLR7 deficiency is predicted to be the least penetrant (inability of pDCs to produce type I and III IFNs). TLR7 deficiency has already been shown to have a high, but incomplete, penetrance (Asano et al., 2021). Only one 5-yr-old child among the young relatives of the 12 index cases carried the LOF TLR7 variant; he remained asymptomatic upon SARS-CoV-2 infection (Fig. 1 C; Asano et al., 2021). Our findings here are thus consistent with predictions and previous reports.

Age itself is probably a major determinant of the penetrance of inborn errors, including the 15 recessive inborn errors studied here (Brodin, 2022). In populations naive for SARS-CoV-2, age is the major epidemiological risk factor for hospitalization or death from pneumonia, with the risk doubling every 5 yr of age, from childhood onward (O’Driscoll et al., 2021). The pediatric population is, therefore, generally considered “safe,” with an IFR of ∼0.001%, and a frequency of critical pneumonia thought to be on the order of 0.01%, but which remains to be estimated accurately (Knock et al., 2021; Le Vu et al., 2021). The risks of comorbidities and auto-Abs against type I IFNs both increase with age (Bastard et al., 2021a; Manry et al., 2022), whereas the levels of tonic type I IFN immunity in the respiratory tract decrease with age (Loske et al., 2021), and the production of type I IFN by pDCs is stronger in children than in adults (Splunter et al., 2019). These factors may both protect children and contribute to the age-dependent increase in the risk of COVID-19 pneumonia. Further studies are required to decipher the underlying mechanisms, but these findings suggest that both the penetrance and severity of COVID-19 for inborn errors of type I IFN immunity may be lower in children than in adults. It is tempting to speculate that the moderate pneumonia seen in children with TYK2 or TLR7 deficiency may have been severe or critical in adults with the same recessive disorder, whereas the severe pneumonia seen in children with TLR7 deficiency may have been critical in adults.

Our findings and previous reports suggest that impaired type I IFN immunity can underlie life-threatening COVID-19 pneumonia in patients of all ages. Auto-Abs against type I IFNs can be found in children and adults, particularly those >60 yr old. A role for auto-Abs neutralizing type I IFNs in children is attested by the high risk of COVID-19 pneumonia in children with autoimmune polyglandular syndrome type 1 (APS-1; Bastard et al., 2021e). Dominant inborn errors of type I IFN immunity can be found in adults, particularly those <60 yr old (Zhang et al., 2020b). It will be important to determine whether they are also found in children and, if so, at what frequency. Recessive inborn errors are found in adults under the age of 60 yr but are more frequent in children. Children with one of at least five known recessive inborn errors of type I IFN immunity (complete defects of STAT2, IFNAR1, TYK2, TLR7, and TBK1) are at high risk of developing COVID-19 pneumonia, including critical pneumonia. Recessive and complete defects of IRF7 (as suggested by studies of adult patients with critical COVID-19 pneumonia), IFNAR2, STAT1, STAT2, IRF9, and UNC93B1 (as suggested by other viral infections in children) probably predispose children to COVID-19 pneumonia with high penetrance. Penetrance may also be high for complete defects of TLR3, TRIF, and MDA5. Inborn errors of type I IFNs should be considered in children hospitalized for COVID-19. Personalized treatment in the first days of infection, including therapeutic type I IFN (Bastard et al., 2021b; Vinh et al., 2021) and monoclonal antibody therapy (Levy et al., 2021), should be considered in patients with defects upstream and downstream from type I IFN receptors, respectively (Zhang et al., 2022). The human genetic and immunological determinants of disease in other children remain to be discovered. Prime candidate genes include those governing the induction of or the response to type I IFNs (Schneider et al., 2021).

## Materials and methods

### Patients

This study included 112 pediatric patients hospitalized for COVID-19 pneumonia in Belgium, Brazil, the Czech Republic, Egypt, France, Hong Kong, Iran, Italy, Peru, Spain, Switzerland, Turkey, the United Arab Emirates, and the United States. Critical COVID-19 pneumonia was defined as critical disease in a patient with pneumonia, whether pulmonary with high-flow oxygen (>6 liters/min) or mechanical ventilation (CPAP, BIPAP, or intubation), or with septic shock or any other type of organ damage requiring ICU admission. This study also included patients with severe COVID-19 pneumonia, defined as pneumonia in a hospitalized patient requiring low-flow oxygen (<6 liters/min) treatment; moderate COVID-19 pneumonia, defined as pneumonia in a patient not requiring oxygen therapy; and mild COVID-19, defined as mild upper respiratory tract symptoms in a patient without pneumonia (Asano et al., 2021).

Written informed consent was obtained in the country of residence of the patients, in accordance with local regulations, and with institutional review board approval. Experiments were conducted in the United States, Sweden, Singapore, and France, in accordance with local regulations and with the approval of the institutional review board. Approval was obtained from the French Ethics Committee “Comité de Protection des Personnes,” the French National Agency for Medicine and Health Product Safety, the “Institut National de la Santé et de la Recherche Médicale,” in Paris, France (protocol no. C10-13), and the Rockefeller University Institutional Review Board in New York (protocol no. JCA-0700).

### Next-generation sequencing

Genomic DNA was extracted from whole blood. The whole exome (*n* = 802) or whole genome (*n* = 534) was sequenced for all 1,336 patients included, at several sequencing centers, including the Genomics Core Facility of the Imagine Institute (Paris, France), the Yale Center for Genome Analysis (New Haven, CT), the New York Genome Center (New York, NY), the American Genome Center (Uniformed Services University of the Health Sciences Bethesda, MD), and the Genomics Division-ITER of the Canarian Health System sequencing hub (Canary Islands, Spain).

For WES, libraries were generated with the Twist Bioscience kit (Twist Human Core Exome Kit), the xGen Exome Research Panel from Integrated DNA Technologies (IDT xGen), the Agilent SureSelect V6 kit, the Agilent SureSelect V7 kit or the SeqCap EZ MedExome kit from Roche, and the Nextera Flex for Enrichment-Exome kit (Illumina). Massively parallel sequencing was performed on a HiSeq4000 or NovaSeq6000 system (Illumina). For WES analysis, performed at CNAG (Barcelona, Spain), capture was performed with the SeqCap EZ Human Exome Kit v3.0 (Roche Nimblegen) and 100-bp paired-end read sequences were obtained on a HiSeq 2000–4000 platform (Illumina). For the OSR Italian cohort, WES was performed with the Agilent SureSelect V7 kit on a NovaSeq6000 system (Illumina).

For WGS on patients of the Italian cohort (the American Genome Center), genomic DNA samples were dispensed into the wells of a Covaris 96 microTUBE plate (1,000 ng per well) and sheared with a Covaris LE220 Focused ultrasonicator, at settings targeting a peak size of 410 bp (t:78; Duty:18; PIP:450; 200 cycles). Sequencing libraries were generated from fragmented DNA with the Illumina TruSeq DNA PCR-Free HT Library Preparation Kit, according to the manufacturer’s protocol but with minor modifications for automation (Hamilton STAR Liquid Handling System), with IDT for Illumina TruSeq DNA UD Index (96 indices, 96 samples) adapters. Library size distribution was assessed and the absence of free adapters or adapter dimers was checked by automated capillary gel electrophoresis (Advanced Analytical Fragment Analyzer). Library concentration was determined by qPCR with the KAPA qPCR Quantification Kit (Roche Light Cycler 480 Instrument II). Sequencing libraries were normalized and combined as 24-plex pools and quantified as above, before dilution to 2.9 nM and sequencing on an Illumina NovaSeq 6000 with the S4 Reagent Kit (300 cycles) and 151 + 8 + 8 + 151 cycle run parameters. Primary sequencing data were demultiplexed with the Illumina HAS2.2 pipeline, and sample-level quality control was performed for base quality, coverage, duplicates, and contamination (FREEMIX <0.05 by VerifyBamID).

We used the Genome Analysis Software Kit (GATK; v3.4-46 or 4) best-practice pipeline to analyze our WES data (DePristo et al., 2011). We aligned the reads obtained with the human reference genome (hg19), using the maximum exact matches algorithm in the Burrows–Wheeler Aligner (Li and Durbin, 2009). PCR duplicates were removed with Picard tools (http://picard.sourceforge.net). The GATK base quality score recalibrator was applied to correct sequencing artifacts. Genotyping was performed with GATK GenotypeGVCFs in the interval intersecting all the capture kits ±50 bp. Sample genotypes with a coverage <8×, a genotype quality <20, or a ratio of reads for the least covered allele (reference or variant allele) over the total number of reads covering the position (minor read ratio) <20% were filtered out. We filtered out variant sites that (a) fell in low-complexity or decoy regions, (b) were multiallelic with more than four alleles, (c) had >10% missing genotypes in our cohort, and (d) spanned >15 nucleotides. Variant effects were predicted with the Ensembl Variant Effect Predictor (McLaren et al., 2016) and the Ensembl GRCh37.75 reference database, retaining the most deleterious annotation obtained from Ensembl protein coding transcripts overlapping with RefSeq transcripts.

### Copy number variant detection

We searched for deletions in the 15 genes of interest, using the NGS data and the HMZDelFinder-opt (Bigio et al., 2021) and MANTA (Chen et al., 2016) algorithms.

### Statistical analysis

We performed an enrichment analysis focusing on 15 candidate genes, on our cohort of 112 pediatric patients with COVID-19 pneumonia, and 1,224 children and adults with asymptomatic or paucisymptomatic infection. We considered variants that were predicted to be LOF or missense and had an MAF <0.0001 (gnomAD v2.1.1). We searched for recessive defects by looking at homozygous and compound heterozygous variants as well as rare homozygous deletions. The quality of the read alignments and phase, for potential compound heterozygotes, was reviewed with the Integrative Genomics Viewer (Robinson et al., 2011). We compared the proportion of patients and controls carrying at least one potential recessive defect in Firth bias-corrected logistic likelihood ratio tests implemented in the logistf R package. In Firth’s regression, a penalty term is assigned to the standard maximum likelihood function used to estimate the parameters of a logistic regression model (Firth, 1993). Firth’s regression can handle genes for which there are no carriers among cases or controls. With no covariates, this corresponds to adding 0.5 to every cell of a 2 × 2 table of allele counts vs. case-control status. We accounted for ethnic heterogeneity by including the first five principal components of the PCA in Firth’s logistic regression model. Analyses were also adjusted for sex. We checked that our adjusted burden test was well calibrated by also analyzing the enrichment in rare (MAF <0.0001) homozygous synonymous variants. We performed PCA with Plink v1.9 software on WES and WGS data, with the 1000 Genomes Project phase 3 public database as a reference, using >15,000 exonic variants with MAF >0.01 and call rate >0.99. We estimated pairwise relatedness between patients and controls using the related option of King software (Manichaikul et al., 2010) and screened for pairs of related individuals, up to the third degree (kinship ≥0.0442). We also estimated the homozygosity rate of the patients and controls from the WES data as the proportion of the autosomal genome in runs of homozygosity (Belkadi et al., 2016). We identified runs of homozygosity with PLINK (Purcell et al., 2007) using a 1,000-kb window and 50 single-nucleotide variations in the window. For this analysis, we used ∼167,000 single-nucleotide variations with a gnomAD frequency >0.05.

### In vitro assays of STAT2 production and function

HEK293T cells were used to seed 6-well plates and were transfected with pCMV-STAT2 WT/mutant variants for 24 h before stimulation with 10,000 U/ml IFN-α2a for 30 min (130-093-874, hIFNa2a; Miltenyi Biotec). The cells were lysed in radioimmunoprecipitation assay buffer, and Western blotting was performed to detect total and phosphorylated STAT2. β-Actin was used as a loading control.

U6A fibrosarcoma cells were transfected with pCMV-STAT2 WT/mutant variants for 24 h before being stimulated with 10,000 U/ml IFN-α2A for 6 h. RNA was extracted with Trizol and the PureLink RNA extraction kit and reverse-transcribed with the Superscript Vilo cDNA production kit. Real-time PCR was performed with the SYBR green kit for IFIT1, IFI27, and RSAD2, with specific primers, and GAPDH was used as the housekeeping gene. Results are expressed according to the ΔΔCt method, where Ct is threshold count, as described by the kit manufacturer.

### Functional assay of IFN responsiveness in human fibroblasts

Primary cultures of human fibroblasts were established from skin biopsy specimens from patients or healthy controls. SV40 fibroblasts were used to seed a 6-well plate and were incubated for 18 h before stimulation with 10,000 U/ml IFN-α2a or IFN-γ for 6 h. The cells were then lysed in radioimmunoprecipitation assay buffer, and Western blotting was performed to detect total and phosphorylated STAT1 and STAT2. GAPDH was used a loading control.

### Luciferase reporter assays for TLR7 functional testing

HEK293T cells, which have no endogenous TLR7 expression, were transfected with the pCMV6 vector bearing WT or variant TLR7 (50 ng), the reporter construct pGL4.32 (100 ng), and an expression vector for *Renilla* luciferase (10 ng), with the X-tremeGENE 9 DNA Transfection Reagent kit (Sigma-Aldrich). The pGL4.32 (luc2P/NF-κB-RE/Hygo; Promega) reporter vector contains five copies of the NF-κB–responsive element (NF-κB-RE) linked to the luciferase reporter gene luc2P. After 24 h, the transfected cells were left unstimulated or were stimulated for 24 h with 1 μg/ml R848 (Resquimod), for activation via TLR7/8 (InvivoGen). Relative luciferase activity was then determined by normalizing the values, using the firefly*:Renilla* luciferase signal ratio.

Western blotting was performed to assess the amounts of protein produced for the TLR7 variants. For whole-cell extracts, the cells were lysed by incubation in the following buffer (50 mM Tris-HCl, pH 8.0, 150 mM NaCl, and 1% NP-40), supplemented with a mixture of protease inhibitors (Sigma-Aldrich), for 30 min at 4°C. The lysates were then centrifuged at 21,000 *g* for 20 min at 4°C. The supernatants were processed directly for Western blotting. Western blotting was performed on 10 µg of total extract from transfected HEK293T cells, with monoclonal antibodies specific for the leucine-rich repeats at the N-terminus of the human TLR7 protein (Cell Signaling Technology) or for amino acid 1,000 at the C-terminus of the human TLR7 protein (Abcam).

### Luciferase reporter assays for MDA5 functional testing

The detailed method has been described elsewhere (Lamborn et al., 2017). In brief, HEK293T cells (80,000 cells/well) were transfected with pIFNB-GL3 (200 ng), pRL-TK (20 ng), and MDA5 or EV (50 ng) at a 1:4 DNA/polyethylenimine ratio and used to seed 96-well plates. The cells were then stimulated, 24 h after transfection, with 2 μg/ml poly(I:C) in the presence of Lipofectamine 2000 (Thermo Fisher Scientific). After stimulation, cells were washed once in PBS and lysed in 200 μl of 1× passive lysis buffer (Promega). Dual luciferase assays were performed on a microplate reader (BMG Labtech Fluostar Omega) in accordance with the manufacturer’s protocol (Promega). Activity was calculated as a percentage of WT MDA5, by dividing the normalized firefly:*Renilla* luciferase ratio for each variant by the normalized WT value and multiplying by 100, and was plotted with Prism 8 software (GraphPad).

### Luciferase reporter assays for IRF7 functional testing

The method has been described in detail elsewhere (Zhang et al., 2020b). HEK293T cells were cotransfected with a mixture of the IFN-β-firefly luciferase reporter plasmid, the pRL-TK-*Renilla* luciferase plasmid, and the pCDNA3-IRF7 plasmid. Cells were incubated for 24 h and were then either left untreated or infected with Sendai virus (20 hemagglutination units/well) for another 24 h. Reporter activity was measured with the Dual-Luciferase Reporter Assay System. Firefly luciferase activity was normalized against *Renilla* luciferase activity. IRF7 expression levels were measured by Western blotting (anti-IRF7 antibody: Cell Signaling; anti-FLAG antibody: Sigma-Aldrich).

### SARS-CoV-2 infection experiments

The SARS-CoV-2 NYC isolate (GenBank accession no. OM345241) was obtained from a de-identified patient. The virus isolate was amplified through 6–7-d passages in Caco-2 cells at 37°C. After each passage, virus-containing supernatant was harvested, clarified by centrifugation (3,000 *g* for 10 min), and filtered through a disposable vacuum filter system with 0.22-μm pores. The passage 3 stock, used in this study, had a titer of 3.4 × 10^6^ PFU/ml determined on Vero E6 cells with a 1% methylcellulose overlay, as described in a previous study (Mendoza et al., 2020). Human SV40 fibroblasts were stably transduced with pTRIP-CD271-2A-ACE2 and positively selected to obtain >90% CD271^+^ cells (130-099-023; Miltenyi Biotec). Cells were used to seed 96-well plates at a density of 7,000 cells per well, with or without 10^3^ IU/ml IFN-α2b (Intron A; MSD). The cells were infected with 0.1 μl/well SARS-CoV-2 16 h later, in 110 μl/well total volume, and were spun for 5 min at 500 *g*. Four replicate infections were performed (separate wells). At 72 hpi, the cells were fixed with neutral buffered formalin at a final concentration of 10%, stained for SARS-CoV-2 with an anti-N-protein antibody at a dilution of 1:3,000 (GTX135357; GeneTex), then with an Alexa Fluor 647–conjugated secondary antibody (A-21245; Invitrogen) and 1 µg/ml Hoechst 33342 (H3570; Invitrogen). Plates were imaged with ImageXpress micro XL and analyzed with MetaXpress (Molecular Devices).

### ISG induction in SARS-CoV-2–infected cells

ISG (IFIT1, MX1, and IFI27) induction was measured by qPCR. In brief, a parallel experiment was performed as described above, except that, rather than staining for immunofluorescence analysis, we lysed the cells and extracted total RNA (Quick-RNA microprep kit; Zimo Research). We then synthesized cDNA with random hexamers (SuperScript III First-strand cDNA synthesis system; Invitrogen). Hs00356631_g1 (Thermo Fisher Scientific), MX1 (Hs00895608_m1; Thermo Fisher Scientific), IFI27 (Hs01086373_g1; Thermo Fisher Scientific), and the housekeeping gene GUSB (β-glucuronidase, 4310888E; Thermo Fisher Scientific) were mixed with cDNA, and their levels were determined (QuantStudio 3 Real-Time PCR system; Applied Biosystems).
